# An outbreak of severe infections among Australian infants caused by a novel recombinant strain of human parechovirus type 3

**DOI:** 10.1038/srep44423

**Published:** 2017-03-14

**Authors:** Tiffanie M. Nelson, Peter Vuillermin, Jason Hodge, Julian Druce, David T. Williams, Rekha Jasrotia, Soren Alexandersen

**Affiliations:** 1 Geelong Center for Emerging Infectious Diseases, Geelong, Victoria 3220, Australia; 2 Deakin University, School of Medicine, Geelong, Victoria 3220, Australia; 3 Barwon Health, University Hospital Geelong, Geelong, Victoria 3220, Australia; 4 Victorian Infectious Diseases Reference Laboratory (VIDRL), Doherty Institute, Melbourne, Victoria 3000, Australia; 5 CSIRO, Australian Animal Health Laboratory, Geelong, Victoria 3220, Australia

## Abstract

Human parechovirus types 1–16 (HPeV1–16) are positive strand RNA viruses in the family *Picornaviridae*. We investigated a 2015 outbreak of HPeV3 causing illness in infants in Victoria, Australia. Virus genome was extracted from clinical material and isolates and sequenced using a combination of next generation and Sanger sequencing. The HPeV3 outbreak genome was 98.7% similar to the HPeV3 Yamagata 2011 lineage for the region encoding the structural proteins up to nucleotide position 3115, but downstream of that the genome varied from known HPeV sequences with a similarity of 85% or less. Analysis indicated that recombination had occurred, may have involved multiple types of HPeV and that the recombination event/s occurred between March 2012 and November 2013. However the origin of the genome downstream of the recombination site is unknown. Overall, the capsid of this virus is highly conserved, but recombination provided a different non-structural protein coding region that may convey an evolutionary advantage. The indication that the capsid encoding region is highly conserved at the amino acid level may be helpful in directing energy towards the development of a preventive vaccine for expecting mothers or antibody treatment of young infants with severe disease.

Picornaviruses are small single-stranded positive sense RNA viruses causing disease in humans and animals including polio in humans and foot-and-mouth disease in ruminants and pigs^1^,^2^. The picornaviruses also include the human parechoviruses (HPeV) that, like other picornaviruses, evolve rapidly due to nucleotide substitutions and recombination events, generating strains associated with changed infectivity, virulence or host range. The first outbreak of HPeV type 3 (HPeV3) detected in Australia occurred in young infants in and around Sydney in New South Wales in late 2013 and a subsequent outbreak occurred across Victoria and Southern Australia between August 2015 – January 2016. The latter outbreak included the first cases of significant illness in infants caused by HPeV seen at University Hospital Geelong and therefore we decided to sequence and further characterise the Geelong 2015 outbreak virus in detail and here report that the causative virus is a novel recombinant strain of HPeV type 3.

HPeV currently consists of 16 types, recently grouped as the species *Parechovirus* A of the genus *Parechovirus* in the family *Picornaviridae*. The other species in this genus, *Parechovirus* B, is also known as Ljungan virus (http://www.picornaviridae.com/parechovirus/parechovirus.htm accessed on 19 September 2016). HPeV type 1 (HPeV1) and 2 were originally classified as echoviruses 22 and 23 in the genus *Enterovirus* but were reclassified in 1998/99 into their own genus on the basis of nucleotide and biological features, including a lack of host cell protein synthesis shut-off during replication^3^,^4^,^5^,^6^,^7^. The RNA genome of the parechoviruses is around 7300–7400 nucleotides and organised into a single long open reading frame (ORF) with 5′ and 3′ untranslated regions (UTR)^8^,^9^,^10^. The polyprotein coding region can be divided into the P1 structural/capsid encoding region and the P2 and P3 non-structural proteins encoding regions. The P1 capsid proteins of the parechoviruses, i.e. both HPeV and Ljungan virus, undergo limited proteolysis and the viral protein 0 (VP0) is somewhat atypical for picornaviruses not cleaved into VP4 and VP2. Consequently, the capsid consists of only 3 proteins, VP0, VP3 and VP1^8^,^11^. For some of the HPeV types the receptor-binding site, likely binding to host cell integrins, is thought to be an arginine-glycine-aspartic acid (RGD) motif near the carboxy-terminus of VP1^12^,^13^. However, this RGD motif is not present in HPeV3 due to a 15 nucleotide indel^8^, nor is it present in HPeV7–16^14^ or in the Ljungan virus^15^,^16^. Strains within certain types normally encoding the RGD motif may also be without it for example for HPeV4^17^ and perhaps also for variant strains of HPeV1 and 6^18^. Like other picornaviruses, recombination of HPeV often involves breakpoints at the very end of VP1 and thus may remove or add the RGD motif, or alternatively occur downstream in the non-structural coding region^8^,^19^,^20^,^21^,^22^,^23^. Studies have indicated that recombinants of HPeV3 with other types may emerge less frequently and suggested that this may be due to a different cell tropism of HPeV3 compared to HPeV1, 2, 4, 5 and 6^8^,^19^,^24^,^25^,^26^,^27^,^28^,^29^.

HPeV are prevalent in humans and have been isolated/detected from both asymptomatic individuals and from patients with a range of clinical features consistent with gastrointestinal, respiratory and central nervous system (CNS) dysfunction. HPeV may cause death among young patients, with one relatively large study reporting mortality from HPeV infection among young infants as high as 6%^30^. HPeV3 may be associated with more severe clinical symptoms particular in very young infants compared to other HPeV types, is the predominant type associated with CNS infection and/or sepsis-like presentations in early infancy and is the most common type identified in cerebrospinal fluid (CSF) samples^12^,^30^,^31^,^32^,^33^,^34^,^35^,^36^,^37^,^38^. The range and severity of clinical manifestations and prognosis of HPeV3 infection is not well defined. In a group of 10 infants with HPeV3 CNS infection, evaluation by cranial magnetic resonance imaging (MRI) or ultrasound showed abnormal periventricular white matter in all infants and in a number of the infants clinical follow-up revealed sequelae including cerebral palsy, epilepsy, learning disabilities, and visual impairment^39^,^40^. Further cases like this are referenced in ref. 18, which also reports detailed findings of autopsy cases of two infants that died with active HPeV3 infection and severe CNS disease.

*In situ* hybridization studies indicate that HPeV3 primarily targets vascular smooth muscle cells in both the leptomeninges and the lung. Thus, clinical symptoms and severe CNS tissue damage may be caused by vascular changes^18^. HPeV3 was first isolated from a faecal sample from a 1-year old patient with transient paralysis in 1999 and reported in the literature in 2004^12^. Subsequent surveillance and testing suggests there is a two or three year cycle between outbreaks^3^,^12^,^31^,^33^,^41^,^42^,^43^. The reason for this temporal pattern is not known, but as these viruses are easily transmitted by the oral-faecal route and by respiratory droplets^35^, it may be related to the pattern of exposure and immunity within families – outbreaks occurring when there is both an older sibling lacking prior HPeV3 exposure plus waning maternally derived HPeV3 passive immunity in the young infant^44^,^45^,^46^. Serological studies from Japan have indicated that antibodies to HPeV3 are only present in 15% of children from 7 months-1 year of age, but that seropositivity increases to 45% in the 2–3 years old and to 85% in the 4–6 years old children^12^. This suggests that strategies to promote humoral immunity against HPeV3 among young infants may be effective - for example, by identification and vaccination of mothers with no or low pre-existing antibody levels to HPeV3^44^. Antibody-mediated neutralization of HPeV3 was initially considered unlikely to be feasible or efficient^47^,^48^, but more recent studies from Japan suggest that these earlier findings may relate to a lack of specific reagents and that antibody neutralization of HPeV3 is efficient^12^,^44^.

Molecular clock analysis estimates that the parechoviruses may have existed for around 400 years and that HPeV3 and HPeV7 diverged around 150 years ago^49^. However, more recent strains appear to have spread worldwide within the last 20–30 years^24^,^33^, and possibly evolved into being more virulent. One such example may be the Yamagata 2008/2014 and the Yamagata 2011 lineages of HPeV3 shown to cause severe disease in young children and myalgia in adults^50^,^51^,^52^.

The first reported outbreak of HPeV3 in Australia took place in and around Sydney in New South Wales (NSW) from October 2013 to January 2014 and overall involved 183 confirmed cases^42^,^43^. Clinical features of sepsis-like disease were reported for 118 infants including poor perfusion, oedema, respiratory distress and a rash, with some infants also having seizures, apnoea and or myoclonic jerks. Only 4% of cases had CSF pleocytosis and the median age at onset of symptoms was 39 days. Approximately 25% of the patients were admitted to an intensive care unit and this group tended to be younger with a median age of onset of symptoms of only 16.5 days (range 4–78 days)^43^. Concurrently, another outbreak of HPeV3 was identified in Melbourne, the capital of Victoria, spanning a longer duration. This outbreak was also observed in other Australian states including Tasmania, South Australia and Western Australia (unpublished). Some of the cases detected in regional Victoria were reported in Ballarat, west of Melbourne in March-May 2014^41^. A subsequent outbreak across Victoria and Southern Australia was detected between August 2015 – January 2016. Here we describe the clinical and molecular virological findings of part of this outbreak in Geelong, a regional center 100 kilometers south of Melbourne, involving 16 cases presenting between August to December 2015. The full 7334 nucleotide genome of the outbreak virus was compared to known sequences and indicated that the outbreak virus was almost 99% similar to the Yamagata 2011 lineage of HPeV3 for the initial 3115 nucleotides of the genome encoding the structural proteins of the virus. Beyond nucleotide 3115, the genome varied from known HPeV sequences with a similarity of 85% or less. Further analysis strongly indicated that recombination had occurred and may have involved more than one type or strain of HPeV. The knowledge gained may be helpful in potential vaccine development or antibody treatment to avoid severe disease in infants and also in understanding evolution, emergence, spread and possibly host range of new/recombinant strains of HPeV3.

## Results

### NGS and Sanger sequencing results

Next generation sequencing was performed on RNA extracted from three clinical samples of CSF, CS-HP-16001–3 (Table 1). The quality of the sequences obtained was highest for CS-HP-16003 followed by CS-HP-16001, while coverage for CS-HP-16002 was relatively poor (Supplementary Table S1). Sample CS-HP-16003 had no coverage for nucleotides 1–158, but good coverage and quality from nucleotide 159–3114 with 4.3 million mapped reads and an average coverage of 68000 (Supplementary Table S1). Sample CS-HP-16001 covered essentially the same nucleotides as sample CS-HP-16003 with a lower number of mapped reads (2.4 mill) and average coverage of 49000. Sample CS-HP-16002 gave only 773000 mapped reads and an average coverage of 1800, however, partial sequences generated were near identical to that observed for CS-HP-16003 and CS-HP-16001. Despite the very good coverage from samples in the P1 region up to genome position 3114, there was relatively little coverage in the latter part of the genome. Limited coverage was indicated in the region around nucleotide 3700–3850, 5300–5400 and 7100–7300 generated from sample CS-HP-16003 and CS-HP-16001.

As the NGS only gave good coverage up to around nucleotide 3114 we suspected that the outbreak virus could be a recombinant with a sequence downstream of that site being very different from the composite reference genome used to generate the Ampliseq panel. Consequently, we designed conventional PCR primers and initially amplified and Sanger sequenced samples CS-HP-16001, 16002 and 16004–16008. This included a fragment from around nucleotide 2700 to 3700 including the potential site of recombination around nucleotide 3100 predicted from the initial NGS sequencing. This Sanger sequencing showed that all 7 clinical samples had a nearly identical sequence (more than 99% identity) and also nearly identical to that obtained for samples 1–3 up to nucleotide 3114 [more than 99% identity and thus same strain and same recombinant around nucleotide 3100]. The next step was to generate Sanger sequence for the parts of the genome missing from the initial NGS and Sanger sequencing, including the very 5′-end of the genome as well as most of the 3′-end of the genome from around nucleotide 3700. For this we selected samples CS-HP-16006 and 16008 as they had shown to contain most virus genome. Additional primers were designed for Sanger sequencing based on the small stretches of sequence obtained from the initial NGS on samples 1 and 3 and the Sanger sequences from samples 16002, 4, 5, 6, 7 and 8. We also used the primers described by Calvert *et al*.^24^ for a segment of 3Dpol. For some areas only sample 16006 was sequenced, however, for all overlaps the sequence was nearly identical to the other samples sequenced (more than 99.5% identity), indicating that all samples tested were the same strain and very closely related/nearly identical. Sequence for the very 5′-terminus was generated using cDNA prepared from the virus isolates (see below).

### Virus isolation

Virus isolation was attempted using CS-HP-16006 fecal and nasal swab samples and CS-HP-16004 fecal sample. No CPE was observed in the first passage (p1) on Vero cells, but significant CPE was observed on day 7 of p2 for all samples, with cell-rounding evident for approximately 25% of monolayers. A more rapid progression of CPE was observed in p3 using filtered p2 supernatants, with 25% CPE observed from day 2 post-inoculation, progressing to 75–100% CPE by day 7, including cell rounding and clearance of the monolayer. We selected the p3 supernatant of the 3 samples and performed partial Sanger sequencing using primers flanking the potential recombination site as well as the primers in the 3Dpol region described by Calvert *et al*.^24^ and primers in the 5′-terminus of the virus genome. This resulted in high quality sequences of all 3 samples covering approximately 780 nucleotides over the potential recombination site and around 730 nucleotides in the 3Dpol region of the genome. The sequences were very similar to/nearly identical to those obtained directly from the clinical samples with variation at the 0.5% level or less. We also obtained 240 nucleotides of high quality sequences from the very 5′terminus that aligned well (more than 99.5% identity) for the samples sequenced and also with the overlap with the consensus sequence obtained from NGS of the initial samples (100% identity at overlap).

### Alignment and assembly of the outbreak parechovirus sequence

Consensus sequence generated from next generation sequencing was exported from IGV and compared to additional sequences generated from Sanger using MEGA-6. Sequences were trimmed outside primer boundaries and aligned to one another using Clustal-W^53^ executed within MEGA-6. Assembly was as follows: Nucleotide 1–174 from Sanger sequencing of the virus isolates (overlap and 100% identity up to nucleotide 240 with that obtained from NGS); nucleotides 175–3114 NGS on CSF samples CS-HP-16003 supported by CS-HP-16001 and for small segments, CS-HP-16002; nucleotides 2624–3767 Sanger sequencing of CSF samples CS-HP-16001, 2, 4, 5, 6, 7 and 8; Sanger sequencing of nucleotide 3590–7154 CSF sample CS-HP-16006; nucleotides 7050–7334 NGS of CSF samples CS-HP-16001 supported by CS-HP-16003 and 2. This overall consensus outbreak strain sequence was further supported by the Sanger sequence obtained from the virus isolates p3 from CS-HP-16004 fecal sample and CS-HP-16006 fecal and nasal swab samples over nucleotides 2718–3498 and 6448–7177 as well as the partial VP1 Sanger sequence from nucleotide 2258–2707 on samples from the 11 cases where this was done (Table 1). The full consensus sequence obtained of the Gelong 2015 HPeV3 outbreak strain has been deposited in GenBank accession number KY020128.

### Analyses of the consensus outbreak strain sequence

Following NGS on samples CS-HP-16001, 2 and 3 we identified a high degree of similarity for the structural coding region of the genome with the Yamagata 2011 lineage of HPeV3^52^. However, we observed little and sporadic coverage from around nucleotide 3100 onwards, and so we suspected this strain was a recombinant. After assembly of the full outbreak strain consensus sequence, we therefore initially compared the region from nucleotide 701 (start of the coding region and the start of the available Yamagata 2011 lineage virus sequences) to 3100 and from nucleotide 3101–7334 separately to all sequences available in the NCBI databases to find the closest matches. For the structural region, the highest levels of similarity (98.7%) was found for the HPeV3 Yamagata 2011 lineage^52^, while for the rest of the genome there was no match greater than 85% sequence similarity.

**Figure 1:**
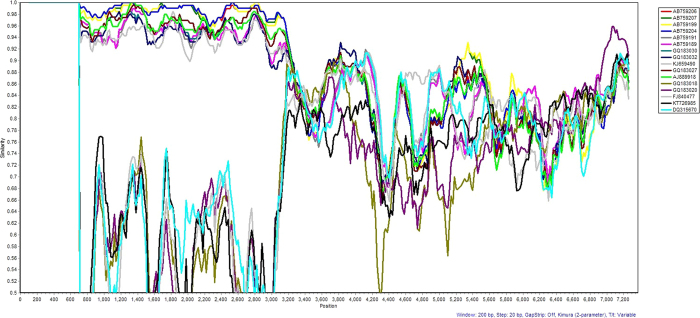
Sequence similarity between Geelong’s outbreak parechovirus and related parechovirus sequences. SimPlot analysis was used to calculate and plot the degree of similarity between the Geelong outbreak parechovirus and a panel of parechovirus strain sequences obtained from NCBI. The key to the panel of strain sequences are as follows: AB759206, AB759207, AB759199, AB759204 HPEV3 (Yamagata 2011 lineage – only include coding regions); AB759189, AB7591 HPeV3 (Yamagata 2008 lineage - only include coding regions); GQ183030, GQ183032, GQ183027 HPeV3; GQ183018, GQ183020 HPEV1; KJ659490 HPeV3; KT726985 HPeV1; AJ889918 HPeV3; FJ840477 HPeV1; and DQ315670 HPeV4. Based on information from AB759206 (HPeV3Yamagata 2011 lineage^52^), the structural/capsid coding region is from nucleotide 701–3013 and the non-structural protein coding region from 3014–7231.

**Figure 2:**
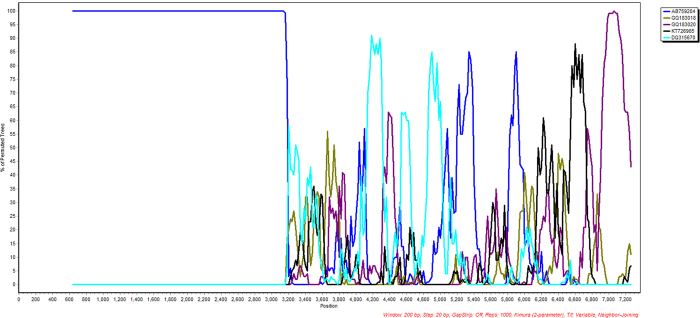
Bootscan analysis of Geelong’s outbreak parechovirus with selected parechovirus sequences. Bootscan analysis was used to calculate the percentage of the permutated tree to which the selected parechovirus sequences cluster to the Geelong outbreak parechovirus sequence. The key to the selected parechovirus sequences is as follows: AB759204 HPeV3 (Yamagata 2011 lineage - only coding region); GQ183018, GQ183020, KT726985 HPeV1; and DQ315670 HPeV4. Based on information from AB759206 (HPeV3Yamagata 2011 lineage^52^), the structural/capsid coding region is from nucleotide 701–3013 and the non-structural protein coding region from 3014–7231.

To further investigate the suspected recombination of this outbreak strain of HPeV3, we compared sequences of the closest matching HPeV sequences in the region downstream of genome position 3100 along with the HPeV3 Yamagata 2011 lineage sequences for similarity using SimPlot and Bootscan^54^,^55^, SBP and GARD analyses^56^,^57^ and the HyPhy package^58^,^59^ available on the Datamonkey webserver^57^,^60^ and using the HKY85 substitution model as suggested by using the automatic Model Selection Tool available on the site. The results of the Simplot and Bootscan analyses (Figs 1 and 2, respectively) indicate a major breakpoint around nucleotide 3116 in the full consensus sequence and are consistent with the SBP and GARD analyses (done on the coding region only). The GARD analysis indicated that further recombination events may have taken place at nucleotides 3115 (essentially identical to the predicted site at 3116), 3815, 5030, 5422 and 6607. However, as no direct closely related sequence downstream of nucleotide 3115 was identified in the NCBI databases, these additional sites may be due to previous recombination events or artifacts caused by the inclusion of several different types and strains into the analysis to cover similarity over the full sequence.

We then compared the segments indicated above to sequences available in the databases at both the nucleotide and amino acid level focusing on the coding region only (nucleotide 701–7231). This comparison showed that the segment from nucleotide 701 to 3115 was 2384/2415(98.7%) identical to HPeV3 Yamagata 2011 (GenBank Accession number: AB759204) at the nucleotide level and was 805/808 (99.6%) identical at the amino acid level. Furthermore, the outbreak strain only had a single conserved change (a Leucine in the outbreak virus and an Isoleucine in the Yamagata 2011 virus) in all of the 758 amino acids encoding the capsid proteins (compared to GenBank Accession number: BAM64940). Comparing the full coding sequence from nucleotide 701–7231 still came up with the Yamagata 2011 as the closest match, however with only 5886/6535(90.1%) identity to the Yamagata 2011 lineage at the nucleotide level, but 2110/2177 (96.9%) at the amino acid level. Comparing the sequence downstream of the indicated site of recombination, i.e. the sequence from nucleotide 3116 to the end of the open reading frame at nucleotide 7231 did not indicate any close match in the NCBI databases but matches around 85% with several sequences deposited from HPeV types 3, 1 and 4, including e.g. 3507/4117(85.2%) to a 2011 type 3 isolate from Taiwan (KT62600). At the amino acid level this part of the genome had the closest match (1312/1369 (95.8%)) to a 2010 type 1 isolate from Taiwan (AMR08586). For completeness, we also compared the 5′- and 3′-UTRs to the NCBI database using Nucleotide BLAST although it should be mentioned that many sequences in the database are not full length and do not include the non-coding regions; that is also the case for the available Yamagata 2011 lineage virus sequences. Nevertheless, the 5′-UTR (nucleotide 1–700) had the closest match (96.7%) to a HPeV3 from Canada in 2001 (AJ88991) while the 3′-UTR (nucleotide 7231–7334) had the highest match (93.3% or 92.4% if excluding the poly-A stretch) to an HPeV1 from China in 2009 (JX441355).

As mentioned above, the GARD analysis indicated potential additional sites of recombination/breakpoints in the non-structural coding region. However, using Nucleotide BLAST comparing the regions indicated, no close ancestors were identified as the region from 3116–3815 was 89% identical or less to the closest matching sequences such as GQ183018 (HPeV1) and GQ183032 (HPeV3); the region from 3816–5030 had 87% or less identity to various HPeV3, 1 and 4 sequences (e.g. GQ183029, FJ840477, DQ315670); 5031–5422 had 90% or less identity to the HPeV3 Yamagata 2011 lineage virus (AB759207); 5423–6607 had 84% or less match to the closest sequences of HPeV6, 4 and 3 while the segment from nucleotide 6608–7231 had 92% identity to HPeV1 (GQ183020).

As no clear ancestor/s to the non-structural coding region could be identified, we attempted further analyses by screening the sequence alignments for potential recombination events using the programs RDP^61^, GENECONV^62^, BOOTSCAN^55^, MAXCHI^63^, CHIMAERA^64^, SISCAN^65^ and 3SEQ^66^ as implemented in the RDP4 software package^67^. Default settings were applied but setting sequences as being linear and the “require topological evidence” and “polish breakpoints” to on and any indels in the alignment treated as single events. To inform the programs about the position of the coding region and that of the individual viral proteins, we used an ORF file from AB759206 (HPeV3Yamagata 2011 lineage^52^). Potential recombination events were considered when three or more methods were in agreement with p-values < 0.001. However, although this detailed analysis for 7/7 methods agreed with our main breakpoint identified above (nucleotide 3115/3116), the specific site suggested was nucleotide 3139 (located in 2A just downstream of the structural coding region), however, the 99% confidence intervals were nucleotide 3053–3216 and it is clearly difficult to pinpoint the exact nucleotide as no close ancestor was available for the non-structural coding region of the outbreak virus. Additional potential regions of recombination were identified in the outbreak virus: nucleotide 3139–4388 (in the 2A and 2C encoding region) and 4570–4775 (2C-3A region) had agreement in 5/7 methods; 4800–5076 (3A) and 5920–7159 (the 3D coding region) had agreement in 4/7 methods; and for nucleotide 5538–5815 (3C coding region) 3/7 methods indicated potential recombination. However, when using Nucleotide BLAST comparing the regions indicated, and as for the GARD analysis, no close ancestors were identified as the regions identified only had a 86–92% match to sequences in the database and included multiple HPeV types and strains including HPEV3, 1, 4, 5 and 6 (e.g. AJ889918, FJ840477, GQ183032, GQ183027, GQ183029, AB759199, AB759191, GQ183020, KJ659490, AB759199).

### Comparison to other selected Australian HPeV3 isolates

The 3 selected Australian virus isolates from 2012, 2013 and 2015 from the VIDRL’s collection were subjected to Sanger sequencing for the regions covering nucleotides approximately 2650–3750 (including the site of recombination) and approximately 6430–7200 in the 3Dpol region with the following results.

VI-HP-16018; this isolate was fully Yamagata 2011-like (not recombinant) based on an overall similarity in the two regions of 1831/1842 (99.4%) identity to Yamagata 2011.

VI-HP-16019 and VI-HP-16020; these isolates were both very similar to the recombinant Geelong 2015 outbreak strain (same recombinant) based on an overall similarity in the two regions of 1895/1905 (99.5% for VI-HP-16019) and 1848/1858 (99.5% for VI-16020) identity to Geelong 2015.

## Discussion

We here describe a recombinant outbreak strain of HPeV3 causing severe clinical disease in infants from August to December 2015 in the city of Geelong, Victoria Australia. The majority of infants were young and the treating teams were compelled to commence empiric systemic antibiotics and admit to hospital for at least 48 hours.

Typing of the HPeV positive cases by Sanger sequencing of a partial VP1 coding region of clinical samples from 11 of the cases showed that the outbreak was caused by a HPeV3 related to the Yamagata 2011 lineage^52^. As this was the first outbreak of HPeV3 observed at University Hospital Geelong and because the Yamagata lineage of HPeV3 is known to cause severe clinical disease^50^,^51^,^52^, we decided to sequence and further characterise the Geelong 2015 outbreak virus in detail. Our initial strategy of using NGS Ampliseq based on a constructed composite full length reference genome containing the coding region from the Yamagata 2011 lineage gave very good coverage for the structural/capsid coding region, but only sporadic coverage downstream of nucleotide 3114 indicating that the Geelong 2015 outbreak virus was likely a recombinant. We therefore performed Sanger sequencing for the other part of the genome on a number of clinical samples and virus isolates. From the Sanger generated sequences together with that generated from NGS, a consensus full-length genome sequence of the Geelong 2015 outbreak HPeV3 was generated consisting of 7334 nucleotides (GenBank Accession number: KY020128). Potential sites and likely ancestors of recombination were then analysed using a multitude of methods and strongly indicated a recombination breakpoint around nucleotide 3115–3139 with 99% confidence intervals for the recombination breakpoint between nucleotides 3053–3216 (located in the 2A non-structural coding region just downstream of the structural coding region). It was not possible to further pinpoint the exact position of the breakpoint as no close ancestor was available for the non-structural coding region. The region from nucleotide 701 to 3115 was 98.7% identical to HPeV3 Yamagata 2011 at the nucleotide level and 99.6% identical at the amino acid level and furthermore, only had a single highly conservative change compared to the Yamagata 2011 virus in all of the 758 amino acids encoding the structural/capsid proteins. Comparing the sequence downstream of the indicated site of recombination, i.e. the sequence from nucleotide 3116 to the end of the open reading frame at nucleotide 7231 did not indicate any close match in the NCBI databases but matches around 85% with several sequences deposited from HPeV types 3, 1 and 4. Since many of the HPeV sequences available for comparison only include the coding region and not the 5′- and 3′-UTRs, we can not at this point determine whether any recombination has taken place involving the UTRs.

Additional potential regions of recombination were identified in the non-structural region of the outbreak virus, however, when comparing the regions indicated, no close ancestors were identified as the regions identified only had 84–92% similarity to the closest matching sequences in the database. Taken together, our results indicated that the outbreak strain was a recombinant with the structural part of the genome derived from and very closely related to the HPeV3 Yamagata 2011 lineage while the region downstream may have derived from a single or multiple recombination events potentially involving several HPeV types with the indication being types 3, 1 and 4 and perhaps also 5 and 6.

In the absence of a proven viral infection, clinicians treating unwell looking febrile young infants are compelled to complete a sepsis work-up, often including lumbar puncture, and commence empirical antibiotics. Antibiotics are then generally continued until the bacterial cultures have failed to isolate a pathogen for at least 48 hours. In this context, access to rapid identification of HPeV infection may reduce the burden of this disease on both the infant and health system by enabling earlier cessation of antibiotics and inpatient care, as well as a decrease in the number of infants requiring a full sepsis work-up. Consequently, development and validation of rapid bedside tests capable of detecting and typing HPeV should be a priority as current methods, employing real time RT-PCR methods for detection and traditional RT-PCR with Sanger sequencing of amplicons for typing, are carried out at centralised laboratories and therefore not capable of providing results to the clinician within a few hours as optimally required. Such development and deployment of rapid bedside tests should be accompanied by extended sequencing efforts performed at centralised laboratories, which importantly should still receive samples, to carefully follow the development of new strains as they may rapidly evolve through recombination as shown by our results.

There is currently no specific treatment for HPeV available. However, at least for HPeV3 that causes the most severe disease in infants, infusion of HPeV3 antibodies early in the development of illness may be effective. Vaccination may also be a viable prevention strategy, potentially targeting mothers with low or absent HPeV3 immunity in order to provide passive immunity to the infant during the first months of life^44^. Therefore, the evidence indicating that the Yamagata 2011 lineage of HPeV3 in the structural region evolves at a rate of around 0.3% per year at the nucleotide level, yet is highly conserved at the amino acid level, as indicated by our findings here, is reassuring in regards to potential development of antibody treatments and preventive vaccines. However, significant recombination has taken place in the non-structural coding region and the effects of this, if any, in regard to the overall ability of this virus to transmit and spread efficiently as well as on host range is currently unknown.

Interestingly, the very first indication of HPeV recombination was reported in 2007 and involved potential recombination between a HPeV4 for the structural region and a HPeV3 for the non-structural region^8^. This is of significance as later studies have indicated that recombinants of HPeV3 may only emerge every 20 years or so, whereas recombinant strains of HPeV1, 2, 4, 5 and 6 appear to emerge roughly every 4 years^19^,^24^,^25^,^26^,^27^,^28^. Some of those studies also looked at sequence variation in the structural region of HPeV1 and HPeV3 and estimated a substitution rate around 0.72% per year for HPeV1 and only 0.28–0.37% per year for HPeV3^19^,^24^. This rate is very similar to what is observed here for the Geelong 2015 outbreak HPeV3 that is around 1.3% different from the Yamagata 2011 HPeV3 in the structural region, consistent with about 4 years between the Yamagata 2011 and the Geelong 2015 outbreaks. However, surprisingly the Geelong 2015 outbreak virus is very different in the non-structural coding region due to recombination with an unknown ancestor/s. The partial Sanger sequencing results of a few selected HPeV3 virus isolates from VIDRL’s collection, suggest that the recombination event may have taken place between March 2012 (a fully Yamagata 2011-like virus isolate from a sample collected in south east Victoria) and November 2013 (a recombinant virus isolate from the first major Australian outbreak of HPeV3 in NSW) and furthermore, that the recombinant HPeV3 strain is remarkably similar when comparing the partial sequences of the NSW 2013 and 2015 isolate with the Geelong 2015 outbreak strain. The origin/source of the genome outside the structural coding region is as mentioned unknown and could simply be due to undetected co-circulating HPeV somewhere in Australia or elsewhere and not yet sequenced and therefore not available in the databases. However, an alternative source could be a yet undiscovered animal/zoonotic reservoir. HPeV4 identical to that seen in humans, and like HPeV3 lacking the RGD receptor motif, has been detected in pigs with anecdotal evidence of neurological disease in Bolivia^17^. The related rodent viruses Ljungan virus and Sebokele virus^68^ and a ferret parechovirus^69^ also lack the RGD motif and Ljungan virus is known to infect and cause disease in people^15^,^16^,^70^,^71^,^72^,^73^,^74^,^75^,^76^. Furthermore, related viruses in the genus *Pasivirus (Parechovirus* sister clade), also lacking the RGD motif have been detected in swine and although apparently not associated with disease in humans, a serosurvey suggested that these or closely-related viruses may infect people^77^,^78^. Other related picornaviruses detected in cattle and sheep in Hungary, and termed hungaroviruses, had features of their 5′ UTR that indicate possible prior recombination with HPeV3^79^. HPeV6 has been detected in monkeys with diarrhoea in China^80^ and HPeV1, 4, 5, 12, 14 and 15 and a Ljungan-related virus detected in monkeys in Bangladesh^81^. Taken together, this may indicate that HPeV, and perhaps in particular HPeV3 characteristically lacking the RGD motif, may also have a zoonotic potential and be able to recombine with related animal/zoonotic viruses. Such interactions are know to occur in picornaviruses; for example the swine pathogen swine vesicular disease virus (SVDV) is thought to have become established in swine by a recombination event/s of the two human picornaviruses coxsackievirus B5 and A9 (now both included in the species *Enterovirus* B) occurring around 1961 and coinciding with large outbreaks of viral meningitis in children in East Asia thought to be where SVDV emerged^82^.

Although we at this point do not know whether this recombinant outbreak virus strain may be more virulent or better able to spread than previously detected strains of HPeV3, further studies are under way to better determine the timing, location and source of the recombination events leading to generation of the Australian 2013/Geelong 2015 outbreak strain of HPeV3 and to better understand the transmission and evolution of this virus at the molecular level. It will be important to establish the detailed mechanisms of generation and selection and the source of this and similar recombinant HPeV3 strains as well as the biological impact and significance of a virus strain with a highly conserved structural shell (capsid essentially 100% conserved at the amino acid level) capable of recombining with and thus obtain highly different non-structural protein encoding regions from other parechoviruses. This ability could potentially lead to a virus with an optimal capsid that by genome recombination has leap-frogged to obtain what may be a near-optimal replication machinery (the genome regions outside the structural protein coding region) and thus providing an evolutionary advantage for infection, replication, excretion, transmission, spread and possibly neurovirulence. Detailed surveillance and analysis of HPeV3 outbreak virus strains over the coming years should be a priority to provide evidence as to whether this particular recombinant does have an evolutionary advantage in regard to transmission, spread and virulence or whether other strains will prevail.

## Materials and Methods

### Geelong regional outbreak and clinical specimens

During August-December 2015 an outbreak of HPeV was identified in infants in the Geelong area of Victoria, Australia. A total of 16 cases, aged between 3 days and 28 weeks (median age 55 days at onset), were identified at the University Hospital Geelong (Table 1 and Supplementary Table S2). Fifteen of the infants presented with fever, which in most cases was associated with irritability, lethargy, poor feeding, respiratory symptoms and a rash. Many also had diarrhoea and/or vomiting. On examination the infants were each unwell looking and most had tachycardia, tachypnoea and a blanching erythematous maculopapular rash. At least one had clinical meningism and another (who was afebrile on presentation) developed seizures that progressed to status epilepticus. In most infants the serum C-reactive protein was either normal or mildly elevated and the full blood examination revealed a mild lymphopaenia. None of the 10 cases where CSF was examined showed pleocytosis. In 15 of the 16 cases the infant’s clinical features were judged to be sufficiently severe to warrant treatment with empirical parenteral antibiotics pending the results of microbial studies (each of the bacterial culture studies remained negative). Antibiotics were not initiated in one case as they had recent exposure to a sibling with proven HPeV. HPeV was detected by PCR of cerebrospinal fluid (CSF) in 10 infants of which 4 also had HPeV detected in their faeces, including one infant with HPeV detected in each of CSF, faeces and nasal swab samples. The remaining 6 infants did not have CSF collected/tested and HPeV was detected in faeces and/or nasal swab samples. Each of the 16 infants recovered from the illness. The studies described here were performed in accordance with relevant guidelines and regulations and was provided with ethical exemption by the Barwon Health Human Research Ethics Committee.

Screening of the samples for human enterovirus and HPeV was performed at the Victorian Infectious Disease Reference Laboratory (VIDRL), Melbourne. RNA was extracted from clinical samples stored in viral transport medium using Qiagen Qiamp 96 and Qiacube HD extraction robotics as per manufacturer’s instructions (Qiagen, Hilden, Germany). RNA was reverse transcribed with random primers using the SensiFAST cDNA Synthesis Kit (Bioline, UK) as per instructions. PCR was performed as previously described for enteroviruses^83^ and for HPeV using a real-time PCR targeting the 5′-UTR with an established in-house protocol. Briefly this in-house HPeV PCR method uses the primers and probe Parecho-F GTTGTAHGGCCCRYGAAGG, Parecho-Ra GTAKYTGGCCCCARATCAGATC, Parecho-Rb GTATCCAGCCCCARATCAGATC and Parecho-MGBProbe FAM-TGCCCAGAAGGTAC-MGBNFQ with 3 μl of cDNA in a final 20 μl fast master mix (Perfecta qPCR fastmix, Quanta Biosciences, MD, USA) containing primers at 0.9 μM and probe at 0.2 μM. Cycling conditions were 95 C for 2 minutes then 45 cycles of 95 C for 2 seconds and 60 C for 30 seconds using an ABI 7500 fast real-time system (Applied Biosystems, CA, USA). The partial VP1 region of selected HPeV-positive samples were PCR-amplified and Sanger sequenced using primers AN353- GACAATAGTTTTGAAATNACNATHCCNTA and AN357- GAATAAAATGGTACTGANARNGTCATYTGYTC described in Nix^84^. Forward and reverse sequences from 11 patients were aligned using MEGA-6^85^ and trimmed to quality reads of 450 nucleotides with only one nucleotide difference among them. Based on these results, sequences were typed as HPeV3 and closely related to the Yamagata 2011 lineage^52^. Extracted RNA or cDNA and clinical specimens were stored at −80 C.

### Next generation sequencing (NGS) of HPeV-positive samples

Based on the HPeV-positive samples with partial VP1 sequences, a composite reference genome from related HPeV3 sequences available in NCBI (National Center for Biotechnology Information) was composed (Supplementary Table S3) and then used to design a custom Ion AmpliSeq Panel (Supplementary Table S4) for use with Ion Torrent S5 System (Thermo Fisher Scientific, Vic. Australia). Extracted RNA converted to cDNA from patient CSF samples CS-HP-16001 to CS-HP-16003 (Table 1) were PCR-amplified with the Ion AmpliSeq Library Kit 2.0 and Ion AmpliSeq Panel. The panel comprises 38 overlapping primer sets (Supplementary Table S4) split into 2 pools per sample and amplified with the 5X Ion AmpliSeq HiFi Master Mix. PCR conditions were as follows: enzyme activation at 99 C for 2 minutes followed by 30 cycles of 99 C for 15 seconds and 60 C for 4 minutes before holding at 10 C. Primer sequences were digested from samples using the FuPa Reagent before barcodes and adaptors were ligated to each sample using Ion Xpress Barcodes 1–3 and Ion P1 Adaptor. Small fragments and impurities were removed from barcoded libraries with the Agencourt AMPure XP system (Beckman Coulter, Lane Cove, NSW, Australia). Libraries were then amplified with primers provided in the library kit for another 5 cycles prior to repeat purification with the AMPure system. All PCRs and incubation steps were conducted on a ProFlex PCR Thermal Cycler (Thermo Fisher). The amplified library was quantified on a High Sensitivity DNA Analysis Kit on the Agilent Bioanalyzer (Agilent Technologies, Santa Clara, CA, USA). Libraries were then pooled prior to loading onto an Ion 530 Chip and loading into the Ion Chef Instrument. Following template preparation, the chip was run on the Ion Torrent S5 System following company protocols. NGS and associated reactions were performed at the Geelong Centre for Emerging Infectious Diseases (GCEID), Geelong, Victoria, Australia.

### Next generation sequence analyses

Sequences obtained from the Ion Torrent S5 were analysed based on each sample having a unique barcode. Raw sequences were analysed using the Ion Reporter Software contained within the Ion S5 Instrument using either the composite reference genome (Supplementary Table S4) or various HPeV reference genomes from NCBI or by *de novo* assembly without a reference using the Assembler plugin^86^,^87^. Obtained reads were then visualised and analysed using the Integrative Genomics Viewer (IGV)^88^ or by comparing obtained contigs to the NCBI database using nucleotide BLAST (Basic Local Alignment Search Tool)^89^,^90^.

### Additional Sanger sequencing

To fill gaps in the assembled genome, we custom designed primers based on the sequences obtained from NGS. As CSF sample CS-HP-16003 was exhausted during the NGS, PCR-amplification was conducted on CS-HP-16001 and CS-HP-16002. RNA was also extracted from HPeV-positive CSF samples CS-HP-16004 to CS-HP-16008. Complementary DNA for the latter was made using the SuperScript VILO cDNA Synthesis Kit (Thermo Fisher) with random hexamers, which was then used for PCR-amplification with primer sets listed in Supplementary Table S5. PCR-amplification was performed using the AmpliTaq Gold 360 Master Mix (Thermo Fisher) with the following conditions: enzyme activation at 95 C for 10 minutes, followed by 40 cycles of 95 C for 30 seconds, 48 C or 54 C for 40 seconds and 72 C for 2 minutes with a final extension of 72 C for 7 minutes. PCR reactions were run on a ProFlex PCR Thermal Cycler (Thermo Fisher). To obtain sufficient products for sequencing, some PCR reactions were further amplified in subsequent PCR reactions using semi-nested PCR with internal primers (Supplementary Table S5). Quantity and quality of PCR products were checked using an Agilent Bioanalyzer followed by gel purification of selected amplicons on 2% Agarose Size Select gels on the E-gel Precast Gel Electrophoresis and Imaging System with appropriately sized DNA marker (Thermo Fisher). Selected amplicons were sequenced with the BigDye Terminator v 3.1 Cycle Sequencing Kit (Thermo Fisher) using the same, or internal, primers used in the PCR reactions. Sequencing products were then purified and prepared using either Agencourt AMPure XP system (Beckman Coulter) prior to the addition of Hi-Di Formamide or by the BigDye XTerminator Purification Kit and then run on a 3500 Series Genetic Analyzer (Thermo Fisher) at the GCEID.

### Virus isolation

For attempts at isolating the virus in cell culture we selected the following 3 samples: CS-HP-16006 fecal and nasal swab samples and CS-HP-16004 fecal sample (Table 1). Each sample was inoculated onto monolayers of Vero cells cultured in 25 cm^2^ flasks to approximately 80% confluence in growth media [EMEM (Invitrogen) supplemented with 10% v/v fetal calf serum (FCS; Serana), 2 mM L-glutamine (Invitrogen), 100 U/ml penicillin (Sigma), 100 μg/ml streptomycin (Sigma), 2.5 μg/ml fungizone (Sigma), and 10 mM HEPES (MP Biomedical)]. Prior to inoculation onto cells, clinical samples were centrifuged at 1200 × *g* for 15 min and the supernatant filtered through a 0.45 μm filter. Sixty microliters of each sample was diluted to 0.5 ml in PBS then inoculated onto cell monolayers. After 1 hour incubation at 37 C, 10 ml of maintenance media (2% v/v FCS) was added and cells were cultured for up to 7 days. Inoculated cell cultures were inspected daily for cytopathic effect (CPE). After 7 days, cultures were frozen at −80 C and thawed 3 times to release cell-associated virus. Harvested supernatants were then clarified by low speed centrifugation (1000 × *g*) before the next passage in cells using 1 ml of clarified supernatant, as above. Three passages were performed for each sample. Following the observation of CPE, pass 2 clarified supernatant was filtered through a 0.22 μm filter prior to pass 3 to confirm the presence of a filterable agent. Virus isolation was conducted at the CSIRO Australian Animal Health Laboratory.

### Australian virus isolates for comparison

Virus sequences from the Geelong 2015 outbreak were also compared to three virus isolates from VIDRL’s collection. These isolates were obtained from clinical samples using methods similar to those described above. The selected virus isolates were:

VI-HP-16018; HPeV-3 isolated from a nasopharyngeal aspirate from a 6 weeks old unwell infant sampled in March 2012 in south east Victoria approximately 20 km from Geelong.

VI-HP-16019; HPeV-3 isolated from CSF from a one week old infant sampled in November 2013 in or close to Sydney in New South Wales (NSW).

VI-HP-16020; HPeV-3 isolated from a faecal sample from a 7 week old unwell infant sampled in November 2015 in or close to Sydney in NSW.

RNA was extracted and converted to cDNA at VIDRL as described for clinical samples and the cDNA was then subjected to partial Sanger sequencing at GCEID as described above.

## Additional Information

**How to cite this article:** Nelson, T. M. *et al*. An outbreak of severe infections among Australian infants caused by a novel recombinant strain of human parechovirus type 3. *Sci. Rep.* **7**, 44423; doi: 10.1038/srep44423 (2017).

**Publisher's note:** Springer Nature remains neutral with regard to jurisdictional claims in published maps and institutional affiliations.

## Supplementary Material

Supplementary Materials

## Figures and Tables

**Table 1:** Sample and patient information.

GCEID Sample ID	Age in weeks	Sample Type	HPeV detected^a^	VP1 sequence available
CS-HP-16001	<1	CSF	P	Yes
CS-HP-16002	6	CSF	P	No
CS-HP-16003	7	CSF	P	Yes
CS-HP-16004	12	CSF	P	Yes
		Faecal	P	No
CS-HP-16005	8	CSF	P	No
CS-HP-16006	4	CSF	P	No
		Faecal	P	No
		Nasal	P	Yes
CS-HP-16007	11	CSF	P	Yes
CS-HP-16008	3	CSF	P	No
CS-HP-16010	9	Faecal	P	No
CS-HP-16011	28	Faecal	P	No
CS-HP-16012	4	CSF	P	No
		Faecal	P	Yes
CS-HP-16013	3	Nasal	P	Yes
CS-HP-16014	15	Nasal	P	Yes
CS-HP-16015	7	CSF	P	No
		Faecal	P	Yes
CS-HP-16016	11	Nasal	P	Yes
		Faecal	P	No
CS-HP-16017	11	Faecal	P	Yes

^a^Reverse-transcription real-time polymerase chain reaction (rRT-PCR) was conducted on patient samples at the Victorian Infectious Disease Reference Laboratory (VIDRL) for diagnostic detection of HPeV and enterovirus. P indicates a positive result for HPeV. All patients shown were positive for HPeV and negative for enterovirus. It should be noted that sample/patient CS-XX-16009 is “missing” in the Table as that sample turned out to be negative for HPeV but positive for enterovirus and therefore not included in the studies described here.
