# Supplementary material for: An outbreak of severe infections among Australian infants caused by a novel recombinant strain of human parechovirus type 3
Source: Sci Rep. 2017 Mar 14;7:44423. doi: 10.1038/srep44423 (PMC5349594; doi:10.1038/srep44423)
Supplement: Supplementary Materials [file srep44423-s1.pdf]

Supplementary materials

Title

An outbreak of severe infections among Australian infants caused by a novel recombinant strain of human parechovirus type 3

Running title

Australian outbreak caused by a recombinant strain of human parechovirus type 3

Authors

Tiffanie M. Nelson[1,2], Peter Vuillermin[2,3], Jason Hodge[1,3], Julian Druce[4], David T. Williams[5], Rekha Jasrotia[3], and Soren Alexandersen[1,2,3*]

[1]Geelong Center for Emerging Infectious Diseases, Geelong, Victoria 3220, Australia
[2]Deakin University, School of Medicine, Geelong, Victoria 3220, Australia
[3]Barwon Health, University Hospital Geelong, Geelong, Victoria 3220, Australia
[4]Victorian Infectious Diseases Reference Laboratory (VIDRL), Doherty Institute, Melbourne, Victoria 3000, Australia
[5]CSIRO, Australian Animal Health Laboratory, Geelong, Victoria 3220, Australia

[*]Corresponding author

Table S1. Next generation sequencing coverage outcomes.

Sample	Number of mapped reads	Average coverage
CS-HP-16001	4.3 million	68000
CS-HP-16002	2.4 million	49000
CS-HP-16003	773000	1800

Table S2. Additional clinical findings

		Clinical features									Laboratory findings			
											**Peripheral blood**			**CSF**
GCEID Sample ID	Age (weeks)	Irritability	Poor feeding	Rash	Fever	Diarrhoea	Tachycardia	Tachypnoea	Coryza	Lethargy	CRP (mg/L)	Neutrophils (10^9/L)	Lymphocytes (10^9/L)	Pleocytosis
CS-HP-16001	<1	+	+	+	+	_	+	+	_	_	<2.9	4.7	4.2	_
CS-HP-16002	6	_	_	_	_	_	+	+	_	_	NC	NC	NC	_
CS-HP-16003	7	+	+	-	+	+	+	-	-	+	<2.9	1.3	6.7	_
CS-HP-16004	12	+	+	+	+	+	+	_	+	_	<2.9	0.6	1.5	_
CS-HP-16005	8	+	+	+	+	_	+	_	+	+	6.6	3.8	2.3	_
CS-HP-16006	4	+	+	+	+	+	+	+	_	_	5.5	2.4	2.2	_
CS-HP-16007	11	+	+	+	+	_	+	_	_	+	11	3.5	2.5	_
CS-HP-16008	3	_	+	+	+	_	+	+	_	+	<2.9	2.0	2	_
CS-HP-16010	9	+	+	-	+	_	+	_	_	_	<2.9	2.7	1.4	-
CS-HP-16011	28	_	+	+	+	+	+	+	+	_	47	2.2	3.4	NC
CS-HP-16012	4	+	+	+	+	_	+	+	+	+	12	2.9	0.3	NT
CS-HP-16013	3	+	+	+	+	+	+	_	+	+	<2.9	3.0	2.4	NC
CS-HP-16014	15	+	+	+	+	_	+	+	_	+	40	3.7	1.7	NC
CS-HP-16015	7	+	+	+	+	+	+	+	+	+	5.4	1.4	2.8	_
CS-HP-16016	11	+	+	+	+	_	+	_	+	_	<2.9	2.1	1.6	NC
CS-HP-16017	11	+	+	+	+	+	+	+	+	+	10	2.5	3.3	NC

CSF = cerebrospinal fluid; CRP = C reactive protein; NC = not collected; NT = specimen collected; + = reported/recorded; - = not reported/recorded

Table S3. Generation of composite reference sequence for design of Ampliseq panel (with partial VP1 sequence from clinical samples)

Nucleotide position	Generated from sequence NCBI ID	Reference
1-57	AJ889918 (1-57)	[38]
58-700	GQ183027 (1-643)	[19]
701-7231	AB759207 (1-6530)	[52]
(2258-2707)	From partial VP1 sequences generated in this study	This study
7232-7334	GQ183027 (7175-7277)	[19]

Based on VP1 sequences generated from clinical samples, a composite reference full-length sequence for construction of an Ampliseq panel was generated with the most closely aligned HPeV sequences at the National Center for Biotechnology Information (NCBI). The VP1 partial sequence generated in this study was 450 nucleotides long, generated from primers AN353 and AN357 designed by [84].

Table S4. Ion Ampliseq Custom Panel Primer Pairs

Amplicon	Forward primer	Reverse primer	Amplicon start	Insert start	Insert stop	Amplicon stop
AMPL1429677	GGGTCTCCTAGAGAGCTTGG	GTACAACACACTTTAAGCGTCTGAAC	9	29	191	217
AMPL1429679	AGATAACAGTTTGCTGCAAAGCATC	CCAACTCACCTAGATAGAGGTAGCT	367	392	608	633
AMPL1429681	GATACTCTTTCCTTGTCTAGTGTGGAAA	CCATTGAATGAAAGTTGTCCACATTTCTAA	746	774	983	1013
AMPL1429683	GCCTATGGTCAAGCCAAATATTTCG	GCAATTGCCCTAGATCAGACGA	1157	1182	1408	1430
AMPL1429684	GGGCTTCTAATGAGGTTGATGTCAC	CACCCACTAGAGCCACAGATTG	1467	1492	1714	1736
AMPL1429686	GCACCACAAAATATGGTGCACA	CAAGTGGAGAAGGAATATGGTATAGTCAAT	1895	1917	2139	2169
AMPL1429688	CCCACTGGATCTTTGGTCTCTTTTC	GTCACATACCAAGCTCTACCAAAGAT	2309	2334	2545	2571
AMPL1429690	GGTTTCTTAGAGTGGCTCATACATATGAC	GGGCACCTTAAACTAATATAAACTTCTGC	2709	2738	2917	2946
AMPL1429692	GGGTTTTTACAAGCACTATGGTATTATTGT	CCAAAAATATCCTTAGCAATGGTTTCACA	3067	3097	3286	3315
AMPL1429694	GGGCTATCTTTACTTGTGCAAAAATGC	CCATCCATTAGACACTGCATGAGT	3461	3488	3684	3708
AMPL1429696	GGGCCTTTTAAGGGTTTTAATGAAGT	TTTTGTCTGGTCTTTTGATTCCACAATAAT	3827	3853	4012	4042
AMPL1429698	AGTTGGCCAGAATACCTTCTAGAATTAGTA	CCTTCCTAGTTTGGCCCAAATCAT	4167	4197	4388	4412
AMPL1429700	GCAAGACTCTGGTGCATTAAAGAGAA	CTGCTGTTTCCAGCAATTATAATTCTTCTG	4558	4584	4771	4801
AMPL1429702	CCTAGACCTAGTGTTTTTAAATGTTTTGCG	CCCTTTGGTTTGGTCACAGGTA	4943	4973	5183	5205
AMPL1429704	ATCAGCATGATGAGATCATTCTACATGG	AACTTCTTTGGTGATAATACCTTGTTCTGT	5334	5362	5575	5605
AMPL1429706	GTGGAAGGAAACTTTAAAATTCTTGGGATG	GCTGCAGACTTCTTGATTAAACACTCA	5723	5753	5970	5997
AMPL1429708	GGTATTGTGGATATGAAAACAGCCATTC	GTTTTCTCAATTCATCTTTAAGGCAAGTGT	6095	6123	6308	6338
AMPL1429710	ATGTTATTACTCAGGCCTTGCTGTT	GCCATGAATTAACCATCTCTCATTGAAGA	6451	6476	6674	6703
AMPL1429712	GATAAAGAAATAGACCCAGAGAAACTGCA	TGGAGGCATAACTCCATTAAGTAGGA	6845	6874	7087	7113
AMPL1429614	CCCTATGTTGCAGACACAAACTATG	GTGGGTTTTGAAAATCCAATTGTAATAAGC	1373	1398	1502	1532
AMPL1429678	CTGGCCGAAGGCAACTAGCAAT	GGCATCTGTTACCAGATGTGAATCC	159	181	402	427
AMPL1429680	CGTAGGTAACAAGTGACACTATGGATCT	CCACCAACTTCATTACCAGAAATAATGTT	570	598	784	813
AMPL1429682	CATCAGCTCCAACTATCCCGTT	TGGACTTGGAAATGAAAGCCACA	951	973	1192	1215
AMPL1429685	ATGAACATGGCCAATGTCCTGA	TTCAAGTTTGGGAATTGGTCTAGTGT	1679	1701	1927	1953

AMPL1429687	TACACAATCTGTGACATTGGTTCAGAA	GGATCTGTAAGGTCCATTTGTGAACC	2102	2129	2344	2370
AMPL1429689	TGTTGACATTTTTACAGTTAGTCACACCA	CCCATTTGAAGACAAGAATGTTTTCCTA	2506	2535	2748	2776
AMPL1429691	GTAGACCAATGATGCATGGGACTA	GCTGTTTTGAAAATGTCATCTGAATCCAAT	2883	2907	3114	3144
AMPL1429693	CTGTCAACTCAGAGCATATCTTCTCAG	CAAAATTTCTGTGGCTGCTGTAGAA	3249	3276	3498	3523
AMPL1429695	GTAACATCTTCTTCAGTGTTGTCACCT	TGTATCCACCAATCAATGTGCCTAA	3647	3674	3863	3888
AMPL1429697	GAGCATGTTTGTGCTATCTTGGATT	CCAGATACCAATTGGTTCCACTCTAATTAA	3977	4002	4207	4237
AMPL1429699	GATGGGTATGACAACCAGGACAT	ATATGCTTTAGCTGCCCTAATATGCATAAT	4355	4378	4594	4624
AMPL1429701	GGACCTAGTCCAGAAAATTACAGAAGATT	TAGCTGCTTTCCTTGTCTGGTTT	4732	4761	4983	5006
AMPL1429703	AAGACTCAAAAGACGAAAGGGCTTA	GTCAAATCTTCTTCCTGTTCAAGGTACT	5148	5173	5372	5400
AMPL1429705	ACTAACAAAATTGGAACAGAAAGCATGT	GGATAGCTACACCCATTTCTCCATTT	5537	5565	5763	5789
AMPL1429707	TGAGCAAATCGGATCCCAGACTA	GTACTCAGGTCCATAGAATTCACGTG	5937	5960	6133	6159
AMPL1429709	GCATTGCTCAAAGGCAATCAGATTTC	GCATTGACCATGAAATGCCAATCTTTATAA	6272	6298	6486	6516
AMPL1429711	GCAACTACACAAACCAGTAATTGACTC	GTTACTTCAGCGCCAAATGAATCTG	6637	6664	6884	6909
AMPL1429713	GGATGAAAAATTTCAGCACATTCAAGC	TTGGTATGTCCAATATTCCAAATTAGTGTTCT	7050	7077	7292	7324

A custom Ion AmpliSeq Panel was generated for use with the Ion Torrent S5 System. Primer sets were designed using the composite HPeV3 reference sequence (**Table S3**).

Table S5. Primers used for PCR-amplification and Sanger sequencing.

Primer sequence	Reference
Forward	
SA-1-26F2: TTTGAAVKKGGTCTCCWAGAGAGCTT	This study
SA-2568-2602F: GCTTGGTATGTGACTTCTCATGATTTTAACAATGG	This study
SA-2640-2668F: GGTCACGGCATGTTATCACAGTTTTTTGC	This study
SA-3063-3097F: GCTTATTTAGATAGGGGTTTTTACAAGCACTATGG	This study
SA-3519-3543F: CCACATTTTTCTCCTCAGCAGCCAC	This study
SA-3541-3580F: CACAGAAATTCTAGATAACGATTTGGTCAAATTCATAGTG	This study
SA-3578-3611F: GTGAAAATACTCGTCAGAATTCTCTGTTACATGG	This study
SA3652-3679F: ATCTTCATCAGTGTTGTCACCCTCCTGC	This study
SA-3682-3708F: GGCACTCATGCAGTGTCTAATGGATGG	This study
SA-3710-3738F: GACGTTAAGAAATTGGCAGAAGTCGTAGC	This study
SA-4961-4995F: GCTTTCATAGAACCAAGACCAAGTGTCTTTAAATG	This study
SA-4995-5023F: GTTTCGCAACTAAAGTTGCAAACCAGACA	This study
SA-5033-5061F: GCTAAGGAAGTTGTGGATTGGTTCAGTAG	This study
SA-5314-5342F: TCTYACACAYTGTGCWGGWTAYCARCAYG	This study
SA-5324-5352F: TGTGCWGGWTAYCARCAYGAKGARATYAT	This study
SA-5342-5370F: CRTGYTGRTAWCCWGCACARTGTGTRAGA	This study
SA-5362-5398F: GCACTCAATTAAGTACCTTGAACAGGMAGAAGATTTG	This study
Calvert-6422-6446F1 GTNTAYARGATGATHATGATGGARA	[24]
Calvert-6512-6534F2 GAYTGGCACTTYATGATYAAYGC	[24]
Reverse	
SA-258-224R: CACCCTGTGTAAAACACAGGTTAGAAAAGAATATG	This study
SA-294-266R: GGTATCTCACTATTAGGGTTATGGCAC	This study
SA-327-304R: GGATGGCTGTGTGAGCATAAGGTC	This study
SA-3805-3768R: ACTGTGTCACAAATTTGTTCTTTGATTTCTTCATCATC	This study
SA-4180-4143R: ACWGTATTGSTAATAWTTTTGTGGCAAATTCTAAAAC	This study
SA-4290-4263R: CCTTGTCCYGGTTTTTCCCGTWTCCAAA	This study
SA-4346-4329R: CACTGGCCGTAGGGGTGG	This study
SA-5342-5314R: GAKGARATYATYYTRCAYGGVCACTCAAT	This study
SA-5352-5324R: ATRATYTCMTCRTGYTGRTAWCCWGCACA	This study
SA-5362-5335R: CCCATGTAGGATAATCTCATCGTGCTGG	This study

SA-5370-5342R: ATTGAGTGBCCRTGYARRATRATYTCMTC	This study
SA-5658-5633R: CCACCCGAATGGTGGACCCTTTGGAC	This study
SA-5738-5706R: GGCCACCACACATTCCTTTACATGATTTAACAG	This study
SA-5801-5771R: CTCCATTTCCAGCTATATGCATTCCAAGAAT	This study
SA-6472-6443R: TGCCAGTCCAGAATAGTAGCATGGAGTTTG	This study
SA-6509-6478R: CCATGAAGTGCCAATCCTTGTATGGGTTAATA	This study
SA 6565-6539R: GGACCCATCATACTGGGAGTAATCCAT	This study
Calvert-7275-7253R1 YTTARTCAACACCATGGGCAYYA	[24]
Calvert-7243-7221R2 ATNACMACWTCATAATCATCCAC	[24]
